# Patients Suffering From Post‐COVID‐19 Syndrome Feature Enhanced Antibody Reactivity Towards Specific Linear Epitopes Within EBV EBNA1

**DOI:** 10.1111/sji.70088

**Published:** 2026-01-10

**Authors:** Peter Lorenz, Felix Steinbeck, Florian Fricke, Franz Mai, Wendy Bergmann‐Ewert, Christine Wossidlo, Emil C. Reisinger, Brigitte Müller‐Hilke

**Affiliations:** ^1^ Institute of Immunology Rostock University Medical Center Rostock Germany; ^2^ Core Facility for Cell Sorting and Cell Analysis Rostock University Medical Center Rostock Germany; ^3^ Division of Tropical Medicine and Infectious Diseases, Center of Internal Medicine II Rostock University Medical Center Rostock Germany

**Keywords:** humoral immunity, linear antibody epitope, PASC post‐acute sequelae of COVID‐19, Post‐COVID‐19 syndrome

## Abstract

Post‐COVID‐19 syndrome (PCS; also known as post‐acute sequelae of COVID‐19, PASC and Long COVID) manifests with various clinical symptoms of unclear aetiology that persist or develop months after acute infection with SARS‐CoV‐2. Potential triggers for PCS include reactivation of latent viruses and autoimmune reactions. In our retrospective cross‐sectional and explorative study we compared 48 PCS patients with 48 individuals that recovered fully from COVID‐19 (convalescents, CC). We focused on characterising humoral immunity by recording IgG antibody reactivity patterns against Epstein–Barr virus (EBV) EBNA1 antigen using peptide microarray and ELISA methodology as well as determining the presence of autoantibodies. The overall binding landscape of IgG antibodies for the EBV EBNA1 protein was similar for the patients with sequelae versus the convalescents. However, the PCS patients displayed stronger reactivity for epitopes contained within the glycine–alanine repeat region of EBNA1, in particular residues 90–325, and within the central part, amino acids 393–420. Intriguingly, in the latter case, the EBNA1 peptide (residues 405–419) that discriminated the PCS and CC cohorts was localised in a different segment C‐terminal from the sequence proposed to be mechanistically associated with multiple sclerosis. The screening for autoantibodies against nuclear/cytoplasmic antigens in HEp‐2 cells and against CRYAB, cardiolipin, beta‐2‐glycoprotein I, IFN‐alpha2, IFN‐omega, and IL‐15 antigens did neither reveal higher prevalence nor increased reactivity in the PCS patients compared to the convalescents. In conclusion, elevated antibody levels against linear peptides derived from residues 90–325 and 405–419 of EBV EBNA1 were the most distinctive characteristics in our cohort of post‐COVID‐19 syndrome patients.

## Graphical abstract

Compared to convalescent individuals, post‐COVID‐19 patients showed increased IgG antibody reactivity in plasma to both a specific central region (amino acids 405–419) and glycine–alanine repeat sequences of EBV EBNA1. The sequences of the central region differ from those associated with molecular mimicry in multiple sclerosis patients.

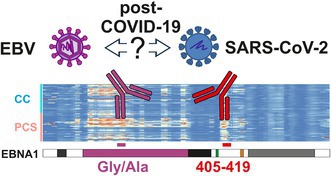

**Abbreviations**
CC: COVID‐19 convalescents
CFS: chronic fatigue syndrome
COVID‐19: coronavirus disease 2019
EBV: Epstein–Barr virus
MS: multiple sclerosis
PCS: post‐COVID‐19 syndrome

## 1 Introduction

The SARS‐CoV‐2 virus causing Coronavirus Disease 2019 (COVID‐19) led not only to a global devastating pandemic but keeps having a tremendous impact on public health with high socio‐economic burden: Many patients report persisting symptoms that cannot be explained by other clinical diagnoses and thus are thought to be causally connected to the previous SARS‐CoV‐2 infection [1, 2, 3]. Such post‐infection disease entities have been coined ‘Long COVID’, ‘post‐COVID‐19 condition/syndrome’ and ‘post‐acute sequelae of SARS‐CoV‐2 infection’ (PASC) [2, 4, 5, 6]. In this manuscript we use the term ‘post‐COVID‐19 syndrome’ (PCS) based on the clinical case condition defined by the World Health Organization as symptom onset within 3 months after SARS‐CoV‐2 infection and persisting for at least 2 months in the absence of alternative medical explanations [7].

Clinically, PCS presents itself as a multifaceted, heterogeneous disorder involving multiple organs and human body systems, including the respiratory, cardiovascular, nervous, gastrointestinal, reproductive and immune systems [5, 8, 9]. A large spectrum of associated symptoms has been reported; most prevalent are post‐exertional malaise, fatigue, brain fog, dizziness and gastrointestinal issues [6]. Diagnosis, stratification, and treatment of patients has been impeded by the lack of well‐defined molecular, physiological, and clinical markers and inconclusive mechanistic models that explain the condition. Patient parameters that increase the likelihood of developing a post‐COVID‐19 condition include age, female sex, pre‐existing health conditions, and frequency and severity of past SARS‐CoV‐2 infections [2, 10]. Current mechanistic hypotheses refer to the possibility of residual reservoirs of SARS‐CoV‐2, organ damage with dysregulated tissue functions as a consequence of infection, perturbation of the immune system including autoimmune‐related pathology, or re‐activation/interaction with existing latent viruses [5, 11, 12, 13].

Epstein–Barr virus (EBV) is one of these long‐term persisting viruses. A role of its reactivation in cells and tissues of COVID‐19 patients for inducing sequelae has been postulated in several publications in recent years [10, 14, 15, 16]. Serological evidence was typically based on increased antibody levels against different EBV proteins, including the EBNA1 antigen. Antibodies against EBNA1 are usually of the IgG type and are quite long‐lived, that is, detectable for many months. Higher anti‐EBNA humoral activity was observed in samples from long/post‐COVID‐19 patients compared to controls [16, 17], but not always [14, 18]. It is not yet clear whether there are mechanistic links between long/post COVID‐19 and EBV. It is possible that the characterisation of the humoral anti‐EBV immune response could help to define subgroups of patients and perhaps establish links to certain persistent COVID‐19 symptoms. It is also conceivable that anti‐EBV EBNA1 antibodies with a certain epitope specificity associated with post‐COVID‐19 could attack the body's own structures in a process known as ‘molecular mimicry’, similar to some autoimmune diseases, in particular MS [11].

The connection of autoimmunity and the post‐COVID‐19 condition has been explored by looking at the humoral arm of acquired immunity and profiling autoantibody specificities. Often, such studies were motivated by the occurrence of certain autoantibodies in association with severe acute COVID‐19. Results reported associations of COVID‐19 sequelae for antibodies against nuclear antigens (ANA) [19, 20, 21], anti‐phospholipid and related antibodies [21, 22] and G protein‐coupled receptors [23]. However, the presence of autoantibodies was not necessarily specifically associated with individuals with post‐COVID‐19 condition [24, 25] or even deemed irrelevant because of their absence [26].

Here, we contribute a study on a cohort of local patients suffering from COVID‐19 sequelae in comparison to convalescents. The study focused on the humoral IgG response against EBV EBNA1 and selected autoantibody specificities against antigens present in HEp‐2 cells, related to thrombotic risk and against the cytokines IFN‐alpha2, IFN‐omega and IL‐15. While the tested autoantibody specificities showed limited utility in distinguishing patients with post‐COVID‐19 syndrome from convalescents, we observed a notable enhancement in IgG reactivity against EBNA1 in patients with sequelae compared to recovered donors. Importantly, specific epitopes within EBNA1 were able to differentiate between the two cohorts, suggesting a potential role for EBV‐directed immunity in post‐COVID‐19 conditions.

## 2 Materials and Methods

### 2.1 Study Participants and Sample Collection

The study follows the ethical principles of the Declaration of Helsinki. The ethics committee of the Rostock University Medical Center approved the study under the file numbers A 2020‐0086 and A 2023‐0081. Written informed consent for participation was obtained from all sample donors.

Patients with post‐COVID‐19 syndrome (PCS) were recruited from the outpatient clinic of the Rostock University Medical Center. Inclusion criteria followed the WHO Delphi Consensus definition (https://www.who.int/publications/i/item/WHO‐2019‐nCoV‐Post_COVID‐19_condition‐Clinical_case_definition‐2021.1) and are based on (i) the confirmation of antibodies recognising the SARS‐CoV‐2 nucleocapsid, (ii) the temporal association between a SARS‐CoV‐2 infection and the onset of characteristic post‐COVID‐19 symptoms, such as fatigue, dyspnea, and cognitive impairment, and (iii) duration of symptoms for at least 2 months. Subjects convalescent for a past COVID‐19 infection were enrolled from clinical, scientific and administrative staff members of the Rostock University Medical Center. All donors in both cohorts, if vaccinated, had a history of vaccination with only RNA vaccines (BioNTech BNT162b2 and/or Moderna mRNA‐1273). The median number of vaccinations was 3 in both groups. However, while the CC group always received at least 3 vaccinations, the PCS cohort contained four patients without any and 8 patients with only 2 vaccinations (*p* = 0.001; Mann–Whitney U‐test). The time interval between sampling and the last recorded vaccination did not differ between the two cohorts (*p* = 0.793; Mann–Whitney U‐test). Blood samples from the PCS cohort were collected between June 2023 and March 2024, those from the CC cohort between January 2023 and February 2024. Plasma was collected by centrifugation after venipuncture in the presence of EDTA as anti‐coagulant and aliquots were stored in an −80°C freezer. The history of infections with SARS‐CoV‐2 was well documented for the CC cohort of health‐care workers with a median number of 2 (range 1–4) and the dates of the first recorded infection between November 2021 and July 2023. For the PCS group, only the infection associated with the post‐COVID diagnosis could be reliably determined. The dates ranged from as early as February 2021 up to March 2023. The overall demographics of the study participants are summarised in Table 1. The people suffering from PCS were subgrouped according to prevalence and severity of symptoms as recorded in their medical documentation. Out of the 48 PCS patients, 11 presented with prominent cardio‐respiratory symptoms (‘CR’), 10 with prominent cognitive impairments (‘CO’) and 4 with fatigue (‘F’) without the first two clinical manifestations. The remaining 23 persons were of a mixed‐type (‘MT’) without a dominating phenotype. The details on each donor, including gender, age, sampling dates, SARS‐CoV‐2‐related PCR and serological data, and selected clinical parameters are documented in Table S1.

**TABLE 1:** Demographics of the study participants.

Cohort	CC	PCS	*p*
*n*	48	48	
Median age in years (min‐max)	51 (26–66)	53 (19–74)	0.570*
Sex (male/female)	14/34	8/40	0.223**

* *p* = (Mann–Whitney *U* test).

** *p* = (Fisher's exact test).

### 2.2 Peptide Microarray

The landscape of the humoral antibody response against overlapping linear peptides derived from Epstein–Barr virus (EBV) EBNA1 protein (Uniprot P03211) was recorded using a commercially available peptide microarray slide (JPT Peptide Technologies GmbH, product RT‐EBV‐EBNA1‐2). One slide carries 21 wells with identical microarrays of 158 peptides in triplicate plus controls. The linear peptides consist of 15‐mers with an additional glycinate residue at the C‐terminal end because of technical reasons during synthesis. Peptides derived from neighbouring sequences in the whole protein overlap by 11 residues. Details of the staining procedure have been described previously [27]. Briefly, the microarrays were incubated with the plasma samples from the donors (diluted 1:200 in blocking buffer SuperBlock/TBS, Thermo Fisher, supplemented with 0.5% Tween 20) for 2 h at 30°C with orbital shaking. After washing with wash buffer (50 mM Tris/HCl pH 7.4, 150 mM NaCl, 0.1% Tween 20), bound primary antibodies were detected with a preparation of goat anti‐human IgG conjugated with Alexa Fluor 647 (Thermo Fisher Z25408, diluted to 0.8 μg/mL in blocking buffer) applied for 1 h at 30°C and orbital shaking. Images were acquired at 5 μm resolution and with 16‐bit depth with a fluorescent scanner (Agilent G2505C) and the signals were evaluated using GenePix Pro version 6.1 (Molecular Devices). The final signals reflect the average fluorescence intensity of the three replicate median foreground fluorescence values for each peptide. Peptide signals that reached at least a fluorescence intensity of 1000 and were simultaneously five times greater than the value of this peptide for buffer instead of sample were designated as ‘specific’. All data are listed in Table S2.

### 2.3 Peptide ELISA

The assay was done as described previously [27]. The peptides were derived from EBV EBNA1 (Uniprot P03211) or the human crystallin alpha B (CRYAB, Uniprot P02511) proteins. Peptide sequences are shown in Table 2 and in the respective data table (Table S3). Peptides (JPT Peptide Technologies GmbH, BioTides) are 15‐mers with an additional C‐terminal glycine acid residue and N‐terminally conjugated with biotin attached to a spacer. They are loaded onto Neutravidin (Thermo Fisher) coated standard ELISA plates. The plasma samples were diluted 1:1000 in antibody dilution buffer (Superblock/TBS plus 0.5% Tween20 and 10 μg/mL Neutravidin). Bound sample antibodies were determined with goat anti‐human IgG antibodies conjugated to peroxidase (Jackson Dianova 109‐035‐088) and subsequent TMB substrate (BioLegend 421101). After stopping the 20 min substrate reaction with 2 N sulfuric acid, the OD450nm values were recorded and plasma‐specific peptide reactivity was obtained by calculating the difference between the raw value and a plasma sample‐specific well without any peptide. If the result was negative, the value was set to zero.

**TABLE 2:** Comparison of plasma antibody reactivity against peptides of interest by peptide microarray and ELISA.

Name	Sequence	Microarray				ELISA		
		Median signal		*p* *	*p* **	Median signal		*p* **
		CC	PCS			CC	PCS	
EBNA1 E‐69	GVRRPQKRPSCIGCK	1275	3105	0.286	0.032	0.572	0.639	0.698
EBNA1 E‐169	AGAGGGAGAGGGAGG	5818	25,577	0.324	0.031	1.845	2.112	0.037
EBNA1 E‐205	GGAGGAGGAGAGGAG	58	178	0.022	0.014	0.078	0.194	0.423
EBNA1 E‐393	SPPRRPPPGRRPFFH	1914	2400	0.663	0.468	0.276	0.332	0.543
EBNA1 E‐401	GRRPFFHPVGEADYF	6994	16,016	0.232	0.018	0.396	0.424	0.435
EBNA1 E‐405	FFHPVGEADYFEYHQ	182	838	0.057	0.008	0.143	0.387	0.015
CRYAB‐3	IAIHHPWIRRPFFPF	n.d.	n.d.	—	—	0.557	0.537	0.98

* Fisher's exact test (fraction of reactive peptides CC vs. PCS).

** Mann–Whitney *U* test (signal intensity distributions CC vs. PCS).

### 2.4 Evaluation of Antibody Reactivity in Plasma Samples Using Commercial Assays

Commercial ELISA assays were used to measure total IgG antibody levels in patient plasma samples against EBV EBNA1 protein (EUROIMMUN EI 2793‐9601 G), EBV early antigen diffuse (EA‐D; EUROIMMUN EI 2795‐9601 G), cardiolipin (EUROIMMUN EA 1621‐9601 G), and beta‐2‐glycoprotein I (EUROIMMUN EA 1632‐9601 G). The presence of IgM‐type antibodies against EBV capsid antigen (CA) was determined with EUROIMMUN EI 2791‐9601 M. The assays were performed as recommended by the manufacturer.

Anti‐cytokine antibodies (anti‐IFN‐alpha2, IFN‐omega, IL‐15) were measured with a magnetic bead based commercial assay in a three‐panel setup (Merck‐Millipore Milliplex HCYTABG‐17 K‐03 assay) using plasma samples diluted at 1:100. Bead signals were read with the Luminex 100/200 system and xPONENT 3.1 software. Sample signals (as mean fluorescence intensity, MFI) were calculated from the raw intensities corrected for the background signal. Readings with less than 50 beads were considered ‘failed’ measurements.

IFN‐alpha2 and IL‐15 cytokine levels were determined with the LEGENDplex bead platform human cytokine panel 2 V02 (BioLegend) as a duplex assay according to the manufacturer's instructions. The readout of the beads was done with the Cytek Aurora flow cytometer. Raw data were analysed with the online software platform available at BioLegend (Data Analysis Software Suite for LEGENDplex). Cytokine concentrations (pg/mL) were calculated with the help of a 7‐point standard curve. Each standard sample was measured twice and averaged; each sample was measured once.

### 2.5 Scoring of Autoantibody Reactivity by Immunofluorescence

Autoantibodies were detected in human HEp‐2 cells (HeLa derivative; European Collection of Authenticated Cell Cultures #86030501), a standard cell line for autoimmune diagnostics, using indirect fluorescence microscopy. Cells grown under standard cell culture conditions on 4‐well Nunc Labtek chamber slides (ThermoFisher 177399) in DMEM (Gibco 31966), 10% fetal calf serum (Sigma S0615), and antibiotics (1 × Pen/Strep, Gibco # 15070) were washed with phosphate‐buffered saline (PBS) and fixed in PBS, 4% (w/v) paraformaldehyde for 15 min. After two washes, cells were permeabilized with PBS, 0.5% (w/v) Triton X‐100 for 5 min. Plasma samples were initially diluted 1:80 in PBS, 1% (w/v) bovine serum albumin, 1% (v/v) normal goat serum, and 0.05% (w/v) Tween 20 (dilution buffer). Cells were incubated with diluted plasma for 30 min, rinsed once, and washed for 10 min with PBS under agitation. Bound human IgG autoantibodies were detected during a 30 min incubation period with goat anti‐human IgG‐AlexaFluor488 (ThermoFisher A11013) secondary antibodies, diluted 1:750 in dilution buffer. After rinsing/washing as described above, cells were counterstained with PBS, 0.5 μg/mL DAPI for 5 min and embedded in mounting medium (Diasorin 1084). All incubations were done at room temperature. Positive plasma samples were further diluted 1:160 and 1:320 for an additional round of the assay. Images were acquired with a Zeiss Axio Observer Z1/7 microscope with a 20 × objective (plan apochromate/NA0.8) and appropriate filters for DAPI and AlexaFluor488. Signals were manually scored by comparing the fluorescence intensity of each sample image to that of a reference image of a positive control sample (ANA‐positive, nucleoli positive; inter‐laboratory test sample), diluted 1:80 and a negative buffer control. The scores reflect the signals of the plasma samples at the 1:80 dilution: (−) signals at background level; (+/−) faint un‐distinguished signal below reference sample fluorescence at 1:320 dilution; (+) signal similar to reference sample fluorescence at 1:320 dilution; (++) signal similar to reference sample fluorescence at 1:160 dilution; (+++) signal similar to reference sample fluorescence at 1:80 dilution.

### 2.6 Computational Tools, Statistics and Figure Preparation

Data processing, heatmap generation as well as some statistical evaluations (Fisher's exact test) were done with packages of the R software environment (www.r‐project.org). Clustering was based on Euclidean distance with complete linkage. Each element in the heatmap reflects the signal of a single peptide. For the heatmap of type ‘Visual map’ we calculated the median signal value of all peptides covering any given AA‐position. One element thus reflects one amino acid. The ‘Visual map’ can be one‐to‐one aligned to the amino acid sequence of a protein. Methods and tools implemented in JASP v 0.19.1 (Sept 09, 2024; https://jasp‐stats.org) were used for statistical evaluation (Mann–Whitney *U* test; Shapiro–Wilk test; binary logistic regression analysis) as well as to draw some box plot illustrations. The statistical significance testing was usually done in a two‐tailed test configuration if not otherwise indicated. In addition, we used the BoxPlotR web‐tool (http://shiny.chemgrid.org/boxplotr/; accessed February 2025) for some box plots. Initial graphs were imported into Corel Draw 2017 (version 19.1.0.419) for post‐processing to get the final figure layout, colouring and additional labelling. Correlations of anti‐peptide signal intensities obtained from peptide chip versus those from peptide ELISA were based on Spearman's rank correlation coefficient (rho and *p*‐value; https://www.wessa.net/rwasp_spearman.wasp). As a resource to find previous studies that reported antibody reactivity against peptides of interest, we interrogated the IEDB immune epitope database (settings: linear peptide, substring, B cell, human, antigen EBV EBNA1; www.iedb.org between March 10 and 18, 2025; [28]). We looked for sequence similarity against human proteins using BLASTp (https://blast.ncbi.nlm.nih.gov/Blast.cgi?PROGRAM=blastp; accessed 2025‐03‐24) within non‐redundant protein sequences from taxid ‘homo sapiens’.

## 3 Results

### 3.1 Antibody Reactivity Landscapes Against EBV EBNA1 Showed Increased Reactivity but Comparable Patterns of PCS Versus CC Plasma Samples

**FIGURE 1:**
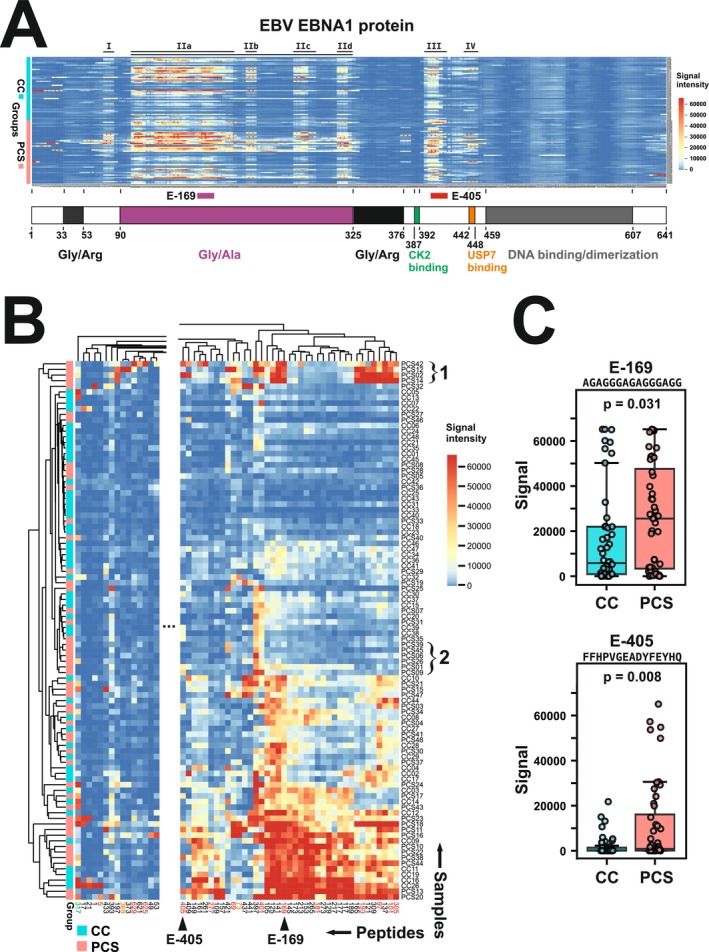
Comparable EBV EBNA1 antibody landscapes in CC and PCS plasma reveal discrete peptide‐level quantitative variations. Results of peptide microarray measurements. (A) ‘Visual map’ of individual EBNA1 peptide microarrays stained with 48 CC and 48 PCS plasma samples. Each line represents one microarray, each row one amino acid. Roman numerals on top of the heatmap label reactivity windows, and the two coloured bars with labels on the bottom depict highlighted peptides. The map is aligned to a schematic drawing of the domain organization of EBNA1 from residues 1 to 641 (according to [29]). The domains include two glycine/arginine‐rich stretches (Gly/Arg), a large glycine/alanine repeat segment (Gly/Ala), binding sites for protein kinase CK2, and Ubiquitin Specific Peptidase 7 (USP7), and the c‐terminal DNA binding and dimerization domain. (B) Excerpt of a heatmap of the clustered (Euclidean distance, complete linkage) data for the CC and PCS samples. Here, each heatmap element represents the reactivity of a sample with a single peptide. The large region that only rarely displayed peptides with signals > 10,000 was cut out for better visibility of more interesting regions. Selected subtree clusters are indicated by curly brackets with numbers to the right. Peptides are designated by the EBNA1 amino residue number they are starting with. Font colours: Peptides' signal distribution reached *p* < 0.05 of Fisher's exact test (green), Mann–Whitney *U* test (red) or for both statistical tests (orange), respectively. The full heatmap is displayed in Figure S1. (C) Higher median antibody reactivity of the PCS compared to the CC cohort for peptides E‐169 and E‐405. Boxplots show the data of the selected peptides (sequence given on top) for both cohorts. The indicated *p*‐values were calculated with a Mann–Whitney *U* test (*N* = 48 for each sample group).

This explorative study aimed to investigate possible differences in linear B‐cell epitopes recognised by IgG‐type antibodies present in plasma samples from people showing persisting disease symptoms after a previous SARS‐CoV‐2 infection (post‐COVID‐19 syndrome, PCS) in comparison to recovered COVID‐19 convalescents (CC). We first focused on using microarrays that carry 158 overlapping linear peptides derived from the EBV EBNA1 protein. All data and statistical evaluations are documented in Table S2. The antibody binding patterns of both study cohorts highlighted several sections of the EBNA1 protein in general that were prone to be antibody targets and did not substantially differ between the cohorts (labelled I‐IV in Figure 1A). The broadest high reactivity window resided between amino acids 101 and 205 with extensions at amino acids 217 to 227, 265 to 287 and 309 to 324 (IIa‐IId). The whole sequence stretch belongs to the glycine–alanine (Gly‐Ala) repeat region of EBNA1 (Figure 1A, bottom). Consequently, the sequences of the different peptides are very similar and some are even identical (Table S2). Towards the N‐terminus of the EBNA1 protein was a narrow signal peak at residues 73 to 83 (region I). In addition, a high reactivity band of peptides was found between amino acids 397 and 419 (region III). This EBNA1 portion lies on the C‐terminal side of residues involved in binding protein kinase CK2 and includes sequences that are known targets of antibodies in the blood of MS patients ([30]; see below). Another sequence stretch with narrow reactivity (IV) was observed between residues 437–451 and contains the binding site of ubiquitin specific peptidase 7 (USP7).

In contrast to these highly reactive stretches, the first approximately 50 residues at the amino end, the segment from 325 to 393 with glycine‐arginine repeats and especially the C‐terminal third of the protein, which covers the entire DNA binding and dimerization domain from amino acid 449 onwards, showed little or only occasional antibody reactivity in only a few samples. The heatmap gave the impression that the PCS and CC samples displayed overall similar antibody reactivity patterns, but that the PCS group might have elevated signals compared to the CC group. When calculating the sum of the signals of all reactive peptides per sample, we indeed found higher cumulative values for the samples of the PCS vs. those from the CC group (median values of PCS 2.2‐fold higher vs. CC samples; *p* = 0.012, Mann–Whitney *U* test).

We next applied clustering to the data and observed that the sample groups were dispersed in a heterogeneous manner, despite small sub‐clusters of just two to maximal six samples of the same cohort on the lowest level of the tree (Figure 1B, for PCS examples see the regions labelled 1, 2 on the right of the heatmap). This indicated the absence of stable qualitative signatures for the PCS and CC cohorts that might have been able to strictly stratify the two groups. For a quantitative comparison of the peptide reactivity of both groups, we determined for each peptide the fraction of samples of each donor group that showed reactivity over background values (‘specific’ peptides) and further compared the exact signal levels between cohorts. In terms of the frequency of ‘specific’ peptides, 7 peptides out of the total of 158 interrogated ones were found to be more often recognised by the PCS samples (*p* < 0.05 significance level, Fisher's exact test). This relative low count supported the notion that PCS as well as the CC samples react with similar linear epitopes of EBNA1. Based on signal intensity, 37 peptides displayed differential reactivity between the cohorts, for most of them (29 peptides) with higher signals from the PCS samples (*p* < 0.05 significance level, Mann–Whitney *U* test; see Table S2). Due to the explorative nature of our study we initially did not perform any correction for multiple testing.

Altogether 18 of the highly reactive peptides with respect to PCS samples reside in the Gly‐Ala repeat region. Given their close sequence homology, similar reactivity was to be expected. The peptide of this group with the highest median reactivity for PCS plasma, E‐169, illustrates the observation of general widespread reactivity with samples from both cohorts but clearly higher antibody reactivity in the PCS group (Figure 1C, top).

The aforementioned EBNA1 protein region with relevance to antibodies in the blood of MS patients contained with E‐401 and E‐405 two overlapping peptides which reached our chosen significance level (*p* < 0.05). Compared to E‐401 (*p* = 0.018), peptide E‐405 appears to be the more selective (*p* = 0.008) and contained a subset of 10 PCS samples (vs. only 1 in case of E‐401) that had higher signals than the maximum value for the CC sample with highest reactivity (E‐405 signal = 21,773; Figure 1C, bottom). In conclusion, we observed a more pronounced anti‐EBNA1 antibody response in PCS patients vs. convalescents while the general patterns of reactivity were comparable.

### 3.2 No Evidence for Active or Recent Infection or Re‐Activation of EBV Virus in the Donors of the PCS and CC Cohorts

**FIGURE 2:**
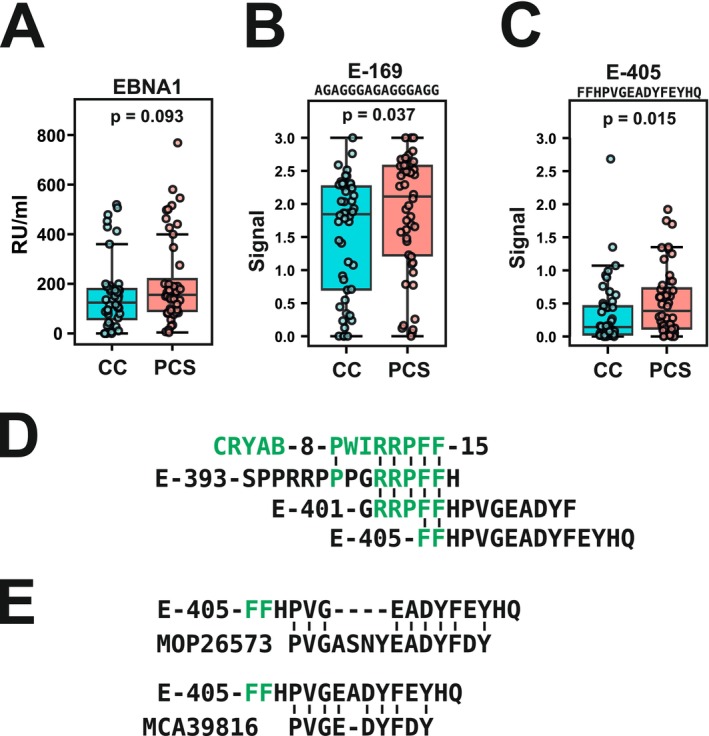
ELISA‐based detection of elevated EBNA1‐directed humoral responses in PCS versus CC individuals. (A) Antibody reactivity (in relative units RU/mL) against the complete EBNA1 protein based on ELISA. (B) Data for peptide E‐169 based on a peptide‐ELISA. Signals reflect background‐corrected OD450nm values. (C) Data as in B for peptide E‐405. The indicated *p*‐values were calculated with a Mann–Whitney *U* test (*N* = 48 for each sample group). (D) Amino acid sequence alignment between a peptide derived from human crystallin alpha B (CRYAB, Uniprot P02511; residues 8–15) and three overlapping peptides from EBV EBNA1 (number indicating the position of the first residue in the whole protein; residues occurring in the CRYAB peptide colored in green). (E) Amino acid alignment of EBNA1 peptide E‐405 with immunoglobulin heavy chain junction sequences (derived from nucleic acid sequences in the indicated GenBank accessions numbers).

To assess the general EBV infection status, we performed serological testing for antibodies against EBV antigens in all samples of the CC and PCS cohorts. None of the samples showed detectable IgM antibodies against EBV virus capsid antigen (CA) and only 1/48 CC or 3/48 PCS samples were positive for IgG‐type antibodies against EBV early antigen‐diffuse (EA‐D) (Table S1). These findings suggest that broad EBV reactivation or novel infection within the weeks to a few months preceding sample collection was unlikely. In contrast, the majority of samples were seropositive for IgG antibodies against EBNA1, indicating a previous EBV infection earlier in their life. The fraction of EBNA1 seropositive samples was slightly higher in the PCS group (45/48) versus the CC cohort (40/48; Fisher's exact test *p* = 0.1986) and the overall signals showed a trend towards higher signals in the PCS versus the CC samples (*p* = 0.093; Mann–Whitney *U* test; Figure 2A). The overall higher reactivity for PSC compared to CC samples using the ELISA with the complete EBNA1 antigen fit the data obtained on the peptide microarray platform above. In summary, our results argue against the presence of active or reactivated EBV at the time of collection of our plasma samples or in the few weeks or months prior to that. Instead, any potential relationship between EBV and the post‐COVID condition should reflect consequences of an EBV infection in the more distant past.

### 3.3 Major Linear EBNA1 Epitopes That Distinguish PCS and CC Samples Reside in the Gly‐Ala Repeat Region and Near, but Outside of a Target Region Related to MS

For validation using a peptide‐ELISA assay, we selected five peptides that distinguished PCS and CC samples in the previous peptide microarray assays. Peptide E‐69 is part of reactivity window I (see Figure 1A). E‐169 and E‐205 were chosen as representatives of the 18 Gly‐Ala repeat‐residing peptides within window II that showed differential signal distributions for PCS and CC. E‐169 was the member of the group with the highest median IgG responses for PCS, and E‐205 showed not only higher median signals but also a higher frequency of positive samples in the PCS cohort (Table 2). The overlapping peptides E‐401 and E‐405 belong to the known target region of IgG antibodies from MS patients (window III). To pinpoint the peak of reactivity of the latter, we also included E‐393, which extends the sequences covered by E‐401/E‐405 peptides N‐terminally but was not found to differentially react with PCS vs. CC samples in the peptide microarray. We then added a peptide from the alpha‐crystallin B chain (CRYAB, residues 3–17, Uniprot P02511) that is thought to be the cellular cross‐reacting antigen in the central nervous system of respective anti‐EBNA1 antibodies because of sequence homology (molecular mimicry [30]; see Figure 2D).

In principle, the peptide‐ELISA data confirmed higher median IgG antibody signals of PCS vs. CC samples for all peptides derived from EBNA1 (Table 2; Table S3). However, differences were mostly very small and signal distributions of the two cohorts were very similar. The data highlighted peptides E‐169 and E‐405 as the only ones that distinguished PCS and CC samples at a statistical significance level below 0.05 (Table 2, Figure 2B,C). The significance of the Spearman correlation between the respective data for the peptide ELISA and the peptide microarray was high (rho = 0.648, *p* < 0.001 for E‐169; rho = 0.435, *p* < 0.001 for E‐405). These two peptides thus fully corroborated the peptide microarray observations.

Interestingly, E‐405 has the least overlap with the CRYAB MS‐related target sequence (Figure 2D). Together with the fact that signal distributions for CRYAB‐3 were indistinguishable for the two sample groups, this argues that the antibodies responsive for higher reactivity in PCS samples are different from those associated with MS in this EBNA1 region. We queried the human protein sequence database at NCBI with the E‐405 amino acid sequence using BLASTp and found the highest homologies in the human proteome with up to 9/14 or 8/10 residues within sequences derived from immunoglobulin heavy chain junction regions (Figure 2E). This result could be coincidental, but it may be worthwhile to further investigate a possible cross‐reactivity of anti‐EBNA1 E‐405 peptide antibodies against immunoglobulins.

### 3.4 Higher Levels of Autoantibodies Against Cytokines IFN‐alpha2 and IL‐15 in Convalescents Compared to Patients With COVID‐19 Sequelae

We were interested whether the presence of autoantibodies distinguished the PCS and CC groups. To get a general overview, we first looked for antibodies in our plasma samples that react with antigens expressed in HEp‐2 cells (ANA anti‐nuclear antibodies; anti‐cytoplasmic antigens) by indirect immunofluorescence. Most samples displayed no or only faint diffuse signals (scoring data in Table S1). Distinctive antibody reactivity to intracellular compartments, in particular the nucleus (ANA‐positive), was observed for 6 samples (12.5%) from the CC versus 10 samples (20.8%) from the PCS cohort (Table 3, scores + and ++; the case +++ did not occur). While the PCS group contained also more samples with strong reactivity (score ++; 4 vs. 1), the overall frequency of autoantibody‐positive samples did not differ (*p* = 0.412, Fisher's exact test).

**TABLE 3:** HEp‐2 autoantibody scores.

Score^a^	CC				PCS			
	All^b^	ANA^b^		Cyt^b^	All^b^	ANA^b^		Cyt^b^
		Chromatin (−)^b^	Chromatin (+)^b^	(+)		Chromatin (−)^b^	Chromatin (+)^b^	(+)
−	14	n.a.	n.a.	n.a.	12	n.a.	n.a.	n.a.
+/−	28	n.a.	n.a.	n.a.	26	n.a.	n.a.	n.a.
+	5	3	1	1	6	4	0	2
++	1	0	1	0	4	1	3	0
+++	0	0	0	0	0	0	0	0

Abbreviation: n.a., not applicable.

^a^ Fluorescence (−) close to buffer control, (+/−) faint and dispersed, (+) similar to reference 1:320, (++) similar to reference 1:160, (+++) similar to reference 1:80.

^b^ Fluorescence patterns of any kind (‘All’), anti‐nuclear antibody‐based (ANA) or in the cytoplasm (Cyt); ANA patterns subcategorized for staining of chromatin (+, −).

Next, we assembled groups based on HEp‐2 autoantibody positivity (scores +, ++ vs. those with ‐, +/−; all CC and PCS samples) and looked at our recorded EBNA1 peptide reactivity as described above in the peptide microarray and ELISA experiments. There was a trend that samples positive for HEp‐2 autoantibodies displayed higher reactivity for peptide E‐169 in the ELISA data compared to the negative samples (*p* = 0.076, Mann–Whitney *U* test). However, in light of a *p*‐value of 0.304 of the same evaluation for E‐169 based on peptide microarray data, postulating a factual connection would be premature.

In the next set of experiments we determined autoantibody levels in plasma in a more targeted manner using assays tailored for specific antigens. Such autoantibodies have been highlighted with respect to severity of COVID‐19 [31]. First, we looked at antibodies associated with thrombotic risk, specifically anti‐ß2 glycoprotein I and anti‐cardiolipin IgG‐type antibodies. None of our samples contained antibodies against ß2 glycoprotein I above the assay cutoff value. Only two samples each from the CC and PCS cohorts had detectable IgG antibodies against cardiolipin (data contained in Table S1). Thus, antibodies against this class of antigens were mostly absent and consequently could not serve as a distinguishing parameter for CC and PCS in our samples.

**FIGURE 3:**
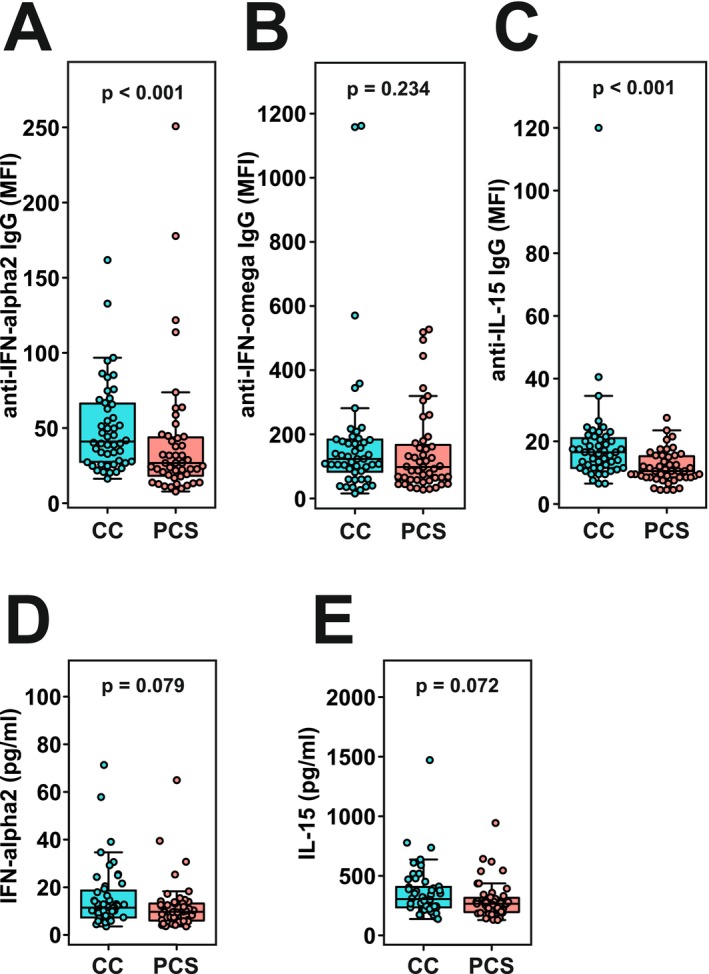
Elevated autoantibody levels against IFN‐alpha2 and IL‐15 in the CC cohort compared to PCS. Autoantibody levels against IFN‐alpha2 (A), IFN‐omega (B), and IL‐15 (C) determined for CC and PCS plasma samples using a magnetic Luminex bead assay. Signals reflect the mean fluorescence intensity of the analytes bound to beads (MFI). The indicated *p*‐values were calculated with a Mann–Whitney *U* test (*N* = 48 for CC, *N* = 47 for PCS cohort). Corresponding plasma cytokine concentrations measured with the LEGENDplexTM bead platform for IFN‐alpha2 (D) and IL‐15 (E). The indicated *p*‐values were again calculated with a Mann‐Whitney U test (N=48 for CC, N=48 for PCS cohort).

With respect to IgG autoantibodies against cytokines, we focused on anti‐IFN‐alpha2, anti‐IFN‐omega, and anti‐IL‐15. Our results indicated that convalescents had significantly higher levels of anti‐IFN‐alpha2 and anti‐IL‐15 IgG antibodies compared to the group with post‐COVID‐19 sequelae (Figure 3 A‐C; *p* < 0.001, Mann–Whitney *U* test; data in Supplement Table S1). To investigate whether this higher autoantibody reactivity coincided with lower cytokine levels, we further determined IFN‐alpha2 and IL‐15 levels in the plasma samples of our two cohorts. There was a trend towards higher IFN‐alpha2 and IL‐15 concentrations in the blood of CC versus PCS samples (Figure 3D,E, *p* = 0.079 and 0.072, respectively). While there was a positive correlation between the levels of both cytokines (Spearman rank correlation coefficient *r* = 0.6534 with *p* = 7.08E‐13), the cytokines did neither show a negative nor a positive correlation with the presence of respective anti‐cytokine autoantibodies (Spearman *r* values and *p*‐value for IFN‐alpha2 versus anti‐IFN‐alpha2 *r* = 0.095/*p* = 0.358; IL‐15 vs. anti‐IL‐15 *r* = 0.122, *p* = 0.237). In addition, neither the anti‐cytokine autoantibody nor the cytokine levels were able to discriminate between HEp‐2‐reactive and non‐reactive plasma samples (IFN‐alpha2 *p* = 0.937, anti‐IFN‐alpha2 *p* = 0.647, IL‐15 *p* = 0.297, anti‐IL‐15 *p* = 0.713, Mann–Whitney *U* test).

Overall, we only found a significant difference in the autoantibodies against the cytokines IFN‐alpha2 and IL‐15, with the values in the CC samples being higher than in the PCS samples.

### 3.5 Association Between E‐169 and E‐405 Antibody Reactivity and PCS Is Not Confounded by Demographic and Clinical Characteristics or Autoantibody Status

Using binary logistic regression analysis, we evaluated the potential influence of confounding factors such as age, sex, the presence of HEp‐2 antibodies and the number of vaccinations on the association of peptide E‐169 and E‐405 reactivity with PCS. Altogether, we observed negligible effects of these factors (Table S4) indicating that they did not confound the association of higher peptide E‐169 and E‐405 reactivity with PCS. The factor ‘number of vaccinations’ even might weaken the initial association since odds ratios increased and *p*‐values decreased in the adjusted model.

In a further analysis, the PCS cohort was categorised according to predominant clinical symptoms (see Methods section). This analysis was constrained by the small and variable sizes of the resulting subgroups. Kruskal‐Wallis tests did not reveal any robust differences in anti‐cytokine antibody reactivity, cytokine levels or antibody responses to peptides E‐169 and E‐405 (Table S5). Likewise, comparisons between peptide‐specific responses and autoantibody profiles (anti‐HEp‐2 or anti‐cytokine reactivity) showed no significant correlations. The comparison of anti‐HEp‐2‐positive versus ‐negative samples yielded *p*‐values of 0.232 and 0.212 for E‐169 and E‐405, respectively (Mann–Whitney *U* test). Spearman rank correlations between anti‐cytokine antibody levels and E‐169 as well as E‐405 peptide reactivity were also weak (Table S6). In summary, neither demographic and clinical variables, nor the measured autoantibodies displayed a robust association with the observed antibody responses, and no parameter effectively stratified the PCS patients beyond the observed group differences.

## 4 Discussion

Our study contributes to the characterisation of the humoral immune response of patients suffering from post‐acute sequelae of SARS‐CoV‐2 infection. We analysed antibodies against linear epitopes in the EBV antigen EBNA1 and a set of autoantibodies. For comparison, we used plasma samples from individuals that fully recovered from a previous COVID‐19 infection.

Antibody epitope mapping of the EBV EBNA1 antigen in the context of the post‐COVID‐19 condition had not been performed previously. Here, we present data that compared the landscape of B cell epitopes residing in linear 15‐mer peptides derived from EBNA1 in PCS and CC cohorts. Our aim was to find potential target sequences in EBNA1 that are specific to patients with post‐COVID‐19 sequelae and ideally contribute to the diagnosis of the disease and/or point to potential cross‐reactive proteins in the human body (‘molecular mimicry’). Overall, our results showed that the patterns of antibody reactivity were very similar in the two cohorts. This implies that the humoral response was not fundamentally different in patients with COVID‐19 sequelae compared to recovered COVID‐19 patients with respect to epitope target space. We found a tendency for higher reactivity of anti‐EBNA1 antibodies in the post‐COVID‐19 samples, but the patterns based on signal strength were not sufficient to enable robust classification of the two cohorts or stratification of subgroups within the PCS patients. Higher antibody reactivity in PCS vs. CC samples towards EBNA1 was observed at a significance level *p* < 0.05 for the sum of all specific peptide signals and for several individual peptides. ELISA results using the full recombinant EBNA1 protein showed a similar trend, although with a slightly reduced level of significance (*p* = 0.093). In the latter case, this difference may be attributable to steric hindrance when epitopes are in close proximity to each other, such as within the glycine–alanine repeat region in EBNA1. A further possibility is the masking of certain sequences within the native protein structure, which may explain the difference to the summed‐up peptide values of the microarray.

In terms of specific targets, peptides E‐169 and E‐405 stood out since both the peptide microarray and the peptide ELISA experiments highlighted stronger signals for the post‐COVID‐19 syndrome samples at significance levels below 0.05. Although the present study was explorative in nature, we sought to strengthen our statistical assessment by applying corrections for multiple testing and analysing effect sizes. Following Benjamini‐Hochberg adjustments, the q‐values for peptides E‐169 and E‐405 were 0.158 and 0.162 for the peptide microarray and 0.130 and 0.105 for the peptide ELISA, respectively. Corresponding effect sizes (Cliff's δ) were 0.313 and 0.257 for the peptide microarray and 0.247 and 0.289 for the peptide ELISA. Although the observed differences between groups were consistent, these q‐values and effect sizes indicate that their statistical strength was modest and the magnitude of the effects should be considered small [32]. Receiver operating characteristic (ROC) analysis supported this interpretation, with areas under the curve ranging between 0.624 and 0.656 for both peptides and both method platforms. Thus, while the identified peptide reactivities reflect genuine differences in humoral immune responses, their capacity to discriminate between PCS and CC samples is limited when considered in isolation.

Peptide E‐169 derives from the glycine–alanine repeat region of EBNA1 and exemplifies the very similar additional 17 peptides from this region that showed increased reactivity with antibodies from post‐COVID‐19 compared to convalescent donors in the peptide microarray experiments. Glycine–alanine repeat antibodies, targeting peptides with the identical or strongly overlapping sequences to E‐169, have been detected most prominently in MS patients. Respective antibodies to the glycine–alanine peptides sometimes distinguished the patients from healthy controls [33, 34], but sometimes not [35]. One study focused on patients with chronic fatigue syndrome (CFS) and was undertaken before the COVID‐19 pandemic [36]. This is of particular interest since CFS is a common symptom among post‐COVID‐19 patients [6]. While a peptide with a 13 residue overlap to our E‐169 sequence displayed a trend towards higher reactivity within that CFS cohort, the significance we have recalculated from the authors' original data was relatively low (*p* = 0.159, Mann–Whitney *U* test; median signal of the samples of the CSF vs. the healthy donor cohort: 2234 vs. 1752, peptide microarray data; [36]). However, since at least a dozen human proteins contain identical matches of at least 10 residues to glycine–alanine repeat sequences of EBNA1 [37], a principle potential for cross‐reactive autoimmune responses remains a valid possibility.

Peptide E‐405 overlaps with a region within EBNA1 that has garnered much attention in relation to autoimmunity. There is evidence that the stretch of EBNA1 protein from residue 386 to 440 contains B‐cell epitopes that resemble similar sequences in human proteins expressed in the central nervous system, namely glial cell adhesion molecule (GlialCAM, EBNA1 residues 393–398), alpha‐crystallin B chain (CRYAB, EBNA1 residues 399–406), and anoctamin 2 (ANO2, EBNA1 residues 431–440). Such ‘molecular mimicry’ is thought to mechanistically link specific anti‐EBNA1 antibodies to MS (reviewed in [38]). Our peptide microarray data showed significantly higher antibody reactivity of the PCS vs. the CC samples against the overlapping E‐401 and E‐405 peptides attributed to the CRYAB sequence homology with the core motif ‘RRPFF’ [30] (see Figure 2D). However, only the E‐405 sequence, which is missing ‘RRP’ and thus three of the 5 amino acids of the motif, could be confirmed with the validation ELISA (see Table 2). At the same time, the antibody signals against the homologous CRYAB peptide itself did not show any difference in signal strength between the 2 cohorts (see Table 2). Thus, B cell reactivity in post‐COVID‐19 patients tends to target linear epitopes within the E‐405 residues that are distinct from the known prominent epitopes in the CRYAB homology region associated with MS.

Antibody reactivity against the identical E‐405 peptide was found in the study on humoral immune responses in CFS patients already mentioned above [36]. As with our glycine–alanine repeat peptide above, our recalculation of those data for E‐405 did again not show a statistically robust association with the CFS condition compared to healthy donors in that study (*p* = 0.139, Mann–Whitney U test). However, given the difference in median signal (13,091 CFS vs. 4629 healthy donor cohort; peptide microarray data), the study of further cohorts may be warranted. In other previously published studies, the E‐405 15‐mer sequence was usually part of longer peptides that extended N‐terminally to include the full CRYAB homology. Higher antibody signal levels against such extended peptides were then associated again with MS in comparison to healthy donors [30, 35]. The E‐405 15‐mer or any substring thereof has not been described as B cell target in the IEDB immune epitope database (www.iedb.org; see Methods) for any other protein than EBNA1.

In conclusion, selected EBNA1‐derived sequences contained epitopes for antibodies with stronger reactivity in patients with COVID‐19 sequelae compared to convalescents that may also reflect specific cross‐reactivity against cellular proteins of the human body. Extended studies with much higher sample count that take PCS subgroups based on different clinical phenotypes into consideration are needed to substantiate this idea. Molecular mimicry is only one possibility of how a previous EBV infection with latently persisting viral states may impact the immune system [39]. Further, the link between post‐COVID‐19 syndrome and EBV infection may be only one example of the general association of viral infections with subsequent chronic conditions [5].

There is evidence that autoimmunity is involved in the pathogenesis of the post‐COVID‐19 condition [11]. The occurrence of autoantibodies is a consequence of a dysregulated immune system with breakdown of tolerance. Autoantibodies likely mediate some of the pathological effects, but their specificities could also be used for stratifying different patient phenotypes. The post‐COVID‐19 patients of our study showed a trend towards a higher prevalence (20.8% against 12.5% positive CC samples) for ANA/cytoplasmic autoantibodies in blood. However, the difference was not statistically robust. In contrast, we observed lower anti‐IFN‐alpha2 and anti‐IL‐15 autoantibodies in the samples of the patients with ongoing disease symptoms compared to the fully recovered persons. The functional consequence is unclear, since the higher anti‐cytokine autoantibody reactivity in the CC group did not coincide with lower concentrations of the respective cytokine. Yet, the presence of such autoantibodies may play a role in homeostasis and/or fine‐tuning of the cytokine response in the healthy condition [40, 41]. Previously published studies found negligible anti‐cytokine autoantibody levels [42] or, in contrast, the association of anti‐IFN‐alpha2 antibodies with the post‐COVID‐19 condition, specifically in patients with respiratory symptoms [10]. A recent report underscored the possibility that cytokine and autoantibody levels in peripheral blood may not necessarily reflect the situation in tissues that are relevant for the post‐COVID‐19 syndrome: Transient IgA‐type anti‐IFN‐alpha antibodies in the nasal epithelium, but not its IgG type counterpart in blood, were correlated with improved recovery from COVID‐19 [43].

The scientific literature contains disparate findings when specifically looking for autoantibody markers for patients with COVID‐19 sequelae. On one hand, highly prevalent IgG type autoantibodies like ANA and against other cellular proteins were found to be associated with gastrointestinal sequelae and phlegm production [10]. In another study, persistent autoantibody reactivity was associated with post‐COVID‐19 syndrome symptoms like fatigue, shortness of breath, and coughing [20]. On the other hand, autoantibody reactivity may be detectable but not be correlated to the post‐COVID‐19 condition [24, 25, 26, 44].

Altogether, it remains unclear whether autoantibodies, that is, autoreactive B cells, play mechanistic roles in the development of the post‐COVID‐19 syndrome. There is discussion of possible pre‐existing conditions involving immune and other factors or direct triggering by the infection with SARS‐Co‐2 may to induce pathological changes. Many intrinsic and extrinsic factors may impact whether infected individuals can resolve the viral infection or suffer long‐term consequences and COVID‐19 sequelae [5, 11]. In a very recently published study, we observed that classical monocytes from patients with PCS express markers of inflammation at lower levels compared to the convalescent control group. This argued for a potential dysregulation of innate immune functions [45].

Our study has several limitations. First, by focusing on 15‐mer peptides, we restricted our analysis to linear epitopes as targets and therefore did not capture antibody specificities against discontinuous epitopes that exist only in the native 3D‐conformation of the antigen. Moreover, our methodology does not account for antibodies directed against post‐translational modifications such as those targeting citrullinated EBNA2 peptides that have diagnostic relevance in rheumatoid arthritis patients [46]. In the context of post‐COVID‐19 syndrome, it remains to be determined whether reactivity towards the identified EBNA1 peptides is functionally linked to molecular mimicry. The discovery of cross‐reactivity with specific cellular proteins may help to delineate mechanisms of immune dysregulation. Future investigations employing natively folded EBNA1 subdomains, together with analyses of potential cross‐reacting host proteins, may help define broader and more conclusive epitope signatures of functional significance. Nonetheless, profiling based on linear peptides can still yield valuable immunological insights. Notably, in the context of EBV and multiple sclerosis, linear 20mer EBNA1‐derived peptides have been shown to provide prognostic value [47]. Our analysis of autoantibody specificities relied on immunofluorescence microscopy of HEp‐2 cells and targeted assays for particular antigens. Thus, we may have missed antibodies against potential targets that are not expressed in these cells and against autoantigens that we did not test for. The nature of our sample collection poses other limitations. Even if the number is comparable with other studies in the field, the sample size of 48 each for PCS and CC cohorts is relatively small. Given the high heterogeneity of post‐COVID‐19 conditions in the absence of definite diagnostic criteria, any stratification may be hard to accomplish. Although the samples have been collected within a similar time period, the histories of infection and vaccination between donors were quite diverse. The CC cohort consisted of participants working in the health care system and thus may have disparate (unknown) properties compared to the recruited PCS patients like health status or healthcare behaviour. We do not know the SARS‐CoV‐2 virus variants underlying the infections of our cohorts. The Omicron variants probably predominated, taking into account the period of enrollment in the years 2023 to 2024. In addition, we only took one snapshot measurement in a cross‐sectional analysis and were not able to look at the dynamics of antibody reactivity in the absence of longitudinal sampling.

In conclusion, our exploratory study presents the first mapping of linear B cell epitopes within the EBV EBNA1 protein for the characterisation of the humoral immune response in the context of post‐COVID‐19 syndrome. A prior EBV infection may contribute to this condition by triggering long‐lasting consequences and alterations of the immune system, for example by reprogramming of the B cells it infects or dysregulation of humoral immunity [48]. However, direct mechanistic links to the Post Covid‐19 condition remain to be established. We identified two antigen segments of particular interest, one distinct from previously reported sequences associated with autoimmunity, which may serve as a starting point for more extended studies and comprehensive mechanistic investigations. Furthermore, we found no significant qualitative or quantitative differences in the examined autoantibody profiles against nuclear and cytoplasmic antigens between patients with COVID‐19 sequelae and convalescents. Taken together, these results underscore the complexity of PCS and highlight the need for biomarkers and mechanistic insights to guide diagnosis, assess risk and inform therapy.

## Author Contributions

Conceptualization: Peter Lorenz, Brigitte Müller‐Hilke; methodology: Peter Lorenz, Felix Steinbeck, Florian Fricke, Franz Mai, Wendy Bergmann‐Ewert, Christine Wossidlo, formal analysis: Peter Lorenz, Felix Steinbeck, Brigitte Müller‐Hilke; writing – original draft preparation: Peter Lorenz; writing – review and editing: Peter Lorenz, Felix Steinbeck, Florian Fricke, Franz Mai, Wendy Bergmann‐Ewert, Christine Wossidlo, Emil C. Reisinger, Brigitte Müller‐Hilke; supervision: Brigitte Müller‐Hilke, Emil C. Reisinger; funding acquisition: Emil C. Reisinger. All authors have read and agreed to the published version of the manuscript.

## Funding

This study was financially supported by the federal State of Mecklenburg‐Western Pomerania, the Ministry of Science, Culture, Federal and European Affairs via the ‘Sondervermögen des MV Schutzfonds, Säule Gesundheit’ GW‐20‐0004. This work was also funded on the basis of a resolution of the German Bundestag by the German government (project ‘COVICare—M‐V’ funding code ZMII2‐2524FSB031).

## Ethics Statement

The study was conducted in accordance with the Declaration of Helsinki, and was approved by the Ethics Committee of the University Medical Center of Rostock (reference numbers A 2020‐0086 and A 2023‐0081).

## Consent

Written informed consent was obtained from all participants involved in the study.

## Conflicts of Interest

The authors declare no conflicts of interest.

## Supporting information


**Figure S1:** Full peptide microarray data for the CC and PCS samples presented as clustered heatmap (Euclidean distance, complete linkage) data. Each heatmap element represents the reactivity of a sample with a single peptide. Note, that only peptides with at least one valid signal have been included (142 of 158 total; Signal > 1000 AND Signal > 5·× buffer background). Selected subtree clusters are indicated by curly brackets with numbers to the right. Peptides are designated by the EBNA1 amino residue number they are starting with. Font colours: Peptides' signal distribution reached *p* < 0.05 of Fisher's exact test (green), Mann–Whitney *U* test (red) or for both statistical tests (orange), respectively.

**Table S1:** Data describing the test subjects (demographics, clinical evaluations) and results from experiments determining anti‐nuclear/anti‐cytoplasmic autoantibodies, anti‐cytokine antibodies and cytokine levels in donor's plasma samples.

**Table S2:** Data table containing the results from the EBV EBNA1 peptide microarray experiments.

**Table S3:** Data table containing the results from the peptide‐ELISA experiments.

**Table S4:** Influence of adjusting for the indicated factors on the association of anti‐EBNA1 peptide reactivity and PCS (binary logistic regression analysis).
**Table S5:** Comparison of antibody reactivity and cytokine levels across PCS symptom‐based subgroups.
**Table S6:** Correlation between peptide antibody and anti‐cytokine reactivity in all samples.

## Data Availability

All data generated during this study are included in this published article (and its Supporting Information in Tables [Link], [Link], [Link]).
