# Supplementary figures and images for: Patients Suffering From Post‐COVID‐19 Syndrome Feature Enhanced Antibody Reactivity Towards Specific Linear Epitopes Within EBV EBNA1
Source: Scand J Immunol. 2026 Jan 10;103(1):e70088. doi: 10.1111/sji.70088 (PMC12789987; doi:10.1111/sji.70088)

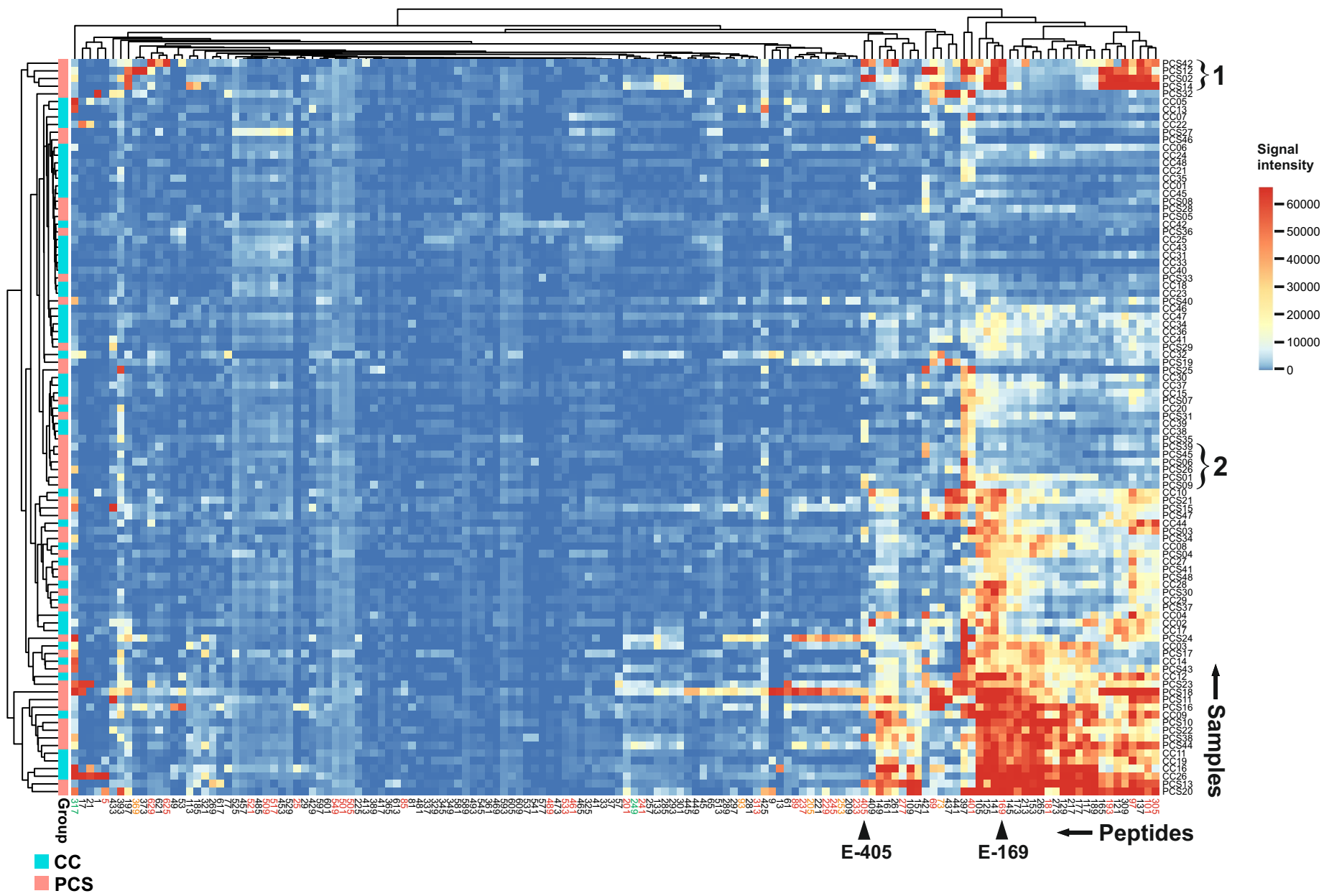

Supplement: Supplementary file 1 — Figure S1: Full peptide microarray data for the CC and PCS samples presented as clustered heatmap (Euclidean distance, complete linkage) data. Each heatmap element represents the reactivity of a sample with a single peptide. Note, that only peptides with at least one valid signal have been included (142 of 158 total; Signal > 1000 AND Signal > 5·× buffer background). Selected subtree clusters are indicated by curly brackets with numbers to the right. Peptides are designated by the EBNA1 amino residue number they are starting with. Font colours: Peptides' signal distribution reached p < 0.05 of Fisher's exact test (green), Mann–Whitney U test (red) or for both statistical tests (orange), respectively. [file SJI-103-e70088-s005.pdf]
