# Supplementary material for: Patients Suffering From Post‐COVID‐19 Syndrome Feature Enhanced Antibody Reactivity Towards Specific Linear Epitopes Within EBV EBNA1
Source: Scand J Immunol. 2026 Jan 10;103(1):e70088. doi: 10.1111/sji.70088 (PMC12789987; doi:10.1111/sji.70088)
Supplement: Supplementary file 5 — Table S4: Influence of adjusting for the indicated factors on the association of anti‐EBNA1 peptide reactivity and PCS (binary logistic regression analysis). Table S5: Comparison of antibody reactivity and cytokine levels across PCS symptom‐based subgroups. Table S6: Correlation between peptide antibody and anti‐cytokine reactivity in all samples. [file SJI-103-e70088-s003.docx]

Manuscript **2924482** Revision 1

**Table S4:** Influence of adjusting for the indicated factors on the association of anti-EBNA1 peptide reactivity and PCS (binary logistic regression analysis)

|  | **Additional factor** | **Without** | **Sex (female; male)** | **Age (years)** | **Age & sex** | **HEp-2 antibodies (yes; no)** | **Vaccinations (n)** |
| --- | --- | --- | --- | --- | --- | --- | --- |
| **E-169** (microarray)* | **OR (95% CI)^#^** | 1.592 (1.041-2.436) | 1.588 (1.033-2.441 | 1.628 (1.059-2.502) | 1.63 (1.055-2.519) | 1.575 (1.027-2.414) | 1.900 (1.179-3.064) |
|  | **p-value^§^** | 0.032 | 0.035 | 0.026 | 0.028 | 0.037 | 0.008 |
|  |  |  |  |  |  |  |  |
| **E-169** (ELISA) | **OR (95% CI)^#^** | 1.514 (0.953-2.403) | 1.552 (0.972-2.478) | 1.5032(0.945-2.389) | 1.538 (0.961-2.461) | 1.465 (0.915-2.343) | 1.755 (1.056-2.917) |
|  | **p-value^§^** | 0.079 | 0.066 | 0.086 | 0.073 | 0.111 | 0.03 |
|  |  |  |  |  |  |  |  |
|  |  |  |  |  |  |  |  |
| **E-405** (microarray)* | **OR (95% CI)^#^** | 3.256 (1.338-7.922) | 3.320 (1.318-8.365) | 3.35(1.407-7.973) | 3.378 (1.384-8.247) | 3.289(1.35-8.015) | 4.187 (1.451-12.085) |
|  | **p-value^§^** | 0.009 | 0.011 | 0.006 | 0.008 | 0.009 | 0.008 |
|  |  |  |  |  |  |  |  |
| **E-405** (ELISA) | **OR (95% CI)^#^** | 2.348 (0.953-5.785) | 2.38(0.975-5.810) | 2.364 (0.956-5.843) | 2.392 (0.977-5.855) | 2.234 (0.895-5.575) | 2.301 (0.928-5.707) |
|  | **p-value^§^** | 0.064 | 0.057 | 0.062 | 0.056 | 0.085 | 0.072 |

***** 16-bit fluorescence signals were z-scaled prior analysis

**^#^** Odds ratio (95% confidence interval)

**^§^** Significance testing using two-sided Wald test

**Table S5: Comparison of antibody reactivity and cytokine levels across PCS symptom-based subgroups.**

**A Count of the subgroups**

| Dominant phenotype | CR | F | CO | MT |
| --- | --- | --- | --- | --- |
| n | 11 | 4 | 10 | 23 |

Syptom type: CR: cardio-respiratory

F fatigue

CO cognitive impairments

MT mixed type

**B Evaluation of differences between subgroups***

|  | H statistic | df | p-value |
| --- | --- | --- | --- |
| E-169 (microarray) | 3.06 | 3 | 0.383 |
| E-169 (ELISA) | 3.386 | 3 | 0.336 |
| E-405 (microarray) | 5.543 | 3 | 0.136 |
| E-405(ELISA) | 0.213 | 3 | 0.975 |
| Peptide microarray (SUM) | 3.354 | 3 | 0.340 |
| EBNA1 (ELISA) | 4.707 | 3 | 0.195 |
| anti-IFNomega | 2.455 | 3 | 0.484 |
| anti-IFNalpha2 | 1.062 | 3 | 0.786 |
| anti-IL-15 | 3.279 | 3 | 0.351 |
| IFNalpha2 | 2.743 | 3 | 0.433 |
| IL-15 | 2.381 | 3 | 0.497 |

* Kruskal-Wallis test

**Table S6: Correlation between peptide antibody and anti-cytokine reactivity in all samples*^#^**

| Variable | anti-IFNomicron | anti-IFNalpha2 | anti-IL-15 |
| --- | --- | --- | --- |
|  |  |  |  |
| E-169 (microarray) | -0.025 (p = 0.808) | -0.030 (p = 0.776) | -0.134 (p = 0.196) |
| E-169 (ELISA) | -0.127 (p = 0.219) | -0.0006 (p = 0.995) | -0.031 (p = 0.768) |
| E-405(microarray) | -0.045 (p = 0.667) | -0.025 (p = 0.814) | -0.087 (p = 0.403) |
| E-405 (ELISA) | 0.003 (p = 0.980) | -0.104 (p = 0.318) | -0.126 (p = 0.222) |

***** n = 96 (48CC + 48 PCS)

**^#^** Spearman’s rho (p-value)
